# Combined experimental and computational characterization of crosslinked collagen-based hydrogels

**DOI:** 10.1371/journal.pone.0195820

**Published:** 2018-04-17

**Authors:** Clara Valero, Hippolyte Amaveda, Mario Mora, Jose Manuel García-Aznar

**Affiliations:** 1 Multiscale in Mechanical and Biological Engineering (M2BE), Aragon Institute of Engineering Research (I3A), Department of Mechanical Engineering, University of Zaragoza, Zaragoza, Spain; 2 Instituto de Ciencia de Materiales de Aragón (CSIC-Universidad de Zaragoza), Departamento de Ciencia y Tecnología de Materiales y Fluidos, University of Zaragoza, Zaragoza, Spain; National University of Ireland - Galway, IRELAND

## Abstract

Collagen hydrogels are widely used for in-vitro experiments and tissue engineering applications. Their use has been extended due to their biocompatibility with cells and their capacity to mimic biological tissues; nevertheless their mechanical properties are not always optimal for these purposes. Hydrogels are formed by a network of polymer filaments embedded on an aqueous substrate and their mechanical properties are mainly defined by the filament network architecture and the individual filament properties. To increase properties of native collagen, such as stiffness or strain-stiffening, these networks can be modified by adding crosslinking agents that alter the network architecture, increasing the unions between filaments. In this work, we have investigated the effect of one crosslinking agent, transglutaminase, in collagen hydrogels with varying collagen concentration. We have observed a linear dependency of the gel rigidity on the collagen concentration. Moreover, the addition of transglutaminase has induced an earlier strain-stiffening of the collagen gels. In addition, to better understand the mechanical implications of collagen concentration and crosslinkers inclusion, we have adapted an existing computational model, based on the worm-like chain model (WLC), to reproduce the mechanical behavior of the collagen gels. With this model we can estimate the parameters of the biopolymer networks without more sophisticated techniques, such as image processing or network reconstruction, or, inversely, predict the mechanical properties of a defined collagen network.

## Introduction

Biological tissues such as bone, skin and organ tissue are of great interest in cellular studies because they are cell natural scaffolds that provide the mechanical microenvironment for cells. Nevertheless, their experimental characterization is quite complicated, presenting a high variability. Therefore, it is not always possible or accessible to work with them. In the last decades, biopolymer hydrogels have become one of the best alternatives to substitute biological tissues in cell culture and tissue engineering [1,2]. Biopolymer hydrogels consist on a polymer network made of connected individual filaments, which are embedded on an aqueous medium. Both properties of the individual filaments and the global structure that they form contribute to the global mechanical properties of the hydrogels. Geometrical parameters such as fiber diameter, network pore size or fiber length may determine the cell behavior inside these networks [3,4] and also the hydrogel mechanical properties [5–7].

It is important to note that the network geometry depends mainly on the hydrogel composition but also on the hydrogel preparation method and the environmental conditions [7–9]. Jones et al. [8] found that modifying the gelation temperature hydrogels also modified their structure and subsequently their mechanical properties. Van Oosten et al. [10] studied the effect of compressing or stretching a biopolymer network after polymerization finding notable differences in the resulting mechanical behavior. Attending to the mechanical behavior, most biopolymers such as collagen or actin are classified as semiflexible polymers, meaning that their bending stiffness can compete with the entropic tendency of forming a random coil [11]. This characteristic induces non-affine deformations in the network, which means that due to bending and stretching, the local deformations are not uniform along the network. Other sources of non-affinity are inhomogeneity in the gel structure induced during preparation and polymerization and network connectivity [12]. Hydrogels are usually viscoelastic and macroscopically incompressible and biopolymer hydrogels present additional properties such as strain-stiffening [13] and negative-normal stress [14].

Mechanical properties of hydrogels also depend on the number of crosslinks that connect the filaments. Fibers can be physically, chemically or enzyme-mediated crosslinked [2]. Biopolymers are naturally physically crosslinked; their fibers form bundles that determine mechanical properties. Physical crosslinks, created by chain entanglement or attraction, are usually weaker than chemical crosslinks [12]. Chemical and enzymatic crosslinking agents can be added during the hydrogel preparation to chemically increase the number of crosslinks. Increasing the crosslink number modifies the network geometry, reducing the distance between joints and creating denser and stiffer networks. Reddy et al. [2] investigated extensively the existing number of crosslinking agents and techniques used for biomedical applications. Although there are many agents that can induce crosslinking in biopolymers, one that has been widely used is glutaraldehyde [15,16]. The main disadvantage of glutaraldehyde is that it can cause cytotoxicity depending on its concentration [17]. Enzymatic crosslinking agents such as lysyl oxidase (LOX) or transglutaminase (TG2) can be naturally found on biological tissues and may be suitable for collagen crosslinking. They perform an important role, but their abnormal activity can induce numerous diseases [18,19]. Transglutaminases are biocompatible crosslinking enzymes, which are naturally found on living organisms [20]. Transglutaminase creates covalent amide bonds between glutamine and lysine. The effect of tissue transglutaminase crosslinked collagen gels has been studied on different cell types such as bone marrow stromal cells [21], dermal fibroblasts [22] and osteoblasts [22,23] finding that the cytotoxicity was under the detection limit. The relative tissue strength of gels crosslinked with different transglutaminase concentration was established through the denaturation temperature [21], finding that crosslinking increases the collagen gel strength. Fortunati et al. [23] also observed higher shear modulus when tissue transglutaminase (TG2) was added to the gels inducing higher cell adhesion, spreading and differentiation. Chau et al [22] reported a slower matrix degradation in the presence of TG2. Nevertheless, the direct effect of transglutaminase on the mechanical properties of collagen gels has not been previously reported to the best of our knowledge.

Collagen hydrogels have been widely studied due to their capacity to mimic biological tissues. Nevertheless, mechanical properties of collagen hydrogels may be measured to assure their similarity to biological tissues. Roeder et al [7] studied the variation of the mechanical properties of collagen hydrogels with different collagen concentration and different pH. They also studied the variation of the linear modulus during hydrogel polymerization, finding that it stabilized after 15 hours. Nevertheless, other studies used various different polymerization times, from minutes [16,24] to 24 hours [25]. A wide study of collagen gel structure was carried by Yang et al [9]. They studied the resulting structure of hydrogels with different collagen concentration finding that fibril density was higher and fibril diameter decreased for higher collagen concentrations. Moreover, they investigated the influence of the gelation temperature finding that when temperature is decreased they visualized fewer filaments but with larger diameter. In addition, different visualization techniques were used and it was found that the use of labeled collagen alters the resulting gelation structures. They also measured greater storage moduli at 22°C than at 37°C. Rheology has been the preferred method to characterize collagen gels in many studies [6,16,25,26], and in other biopolymers [27]. Rheology studies the flow and deformation of matter, which reports the interrelation between force, deformation and time. Rheology not only deals with the deformation of solids and liquids, but also viscoelastic materials that show properties between an ideal solid (elastic) and an ideal liquid (viscous) in response to force, deformation and time. Rheological properties of the substances can be determined using rotational rheometers, among others. Rheology has been recently used to study whether collagen hydrogels mechanics is controlled by stress [28] or strain [26] paying attention to the influence of collagen concentration on the hydrogel stiffness.

To deeply understand biopolymer hydrogels many computational models have been proposed to simulate and evaluate the mechanical properties of hydrogels under various loading modes. These works can be divided in two main groups, continuous and discrete approaches. Among the continuum approaches the worm like chain (WLC) type models have been used to simulate biopolymer behavior [29,30]. Palmer and Boyce [29] combined a WLC model [31] and the Arruda-Boyce [32] constitutive model to reproduce semiflexible polymer behavior. Although they tested the model with actin networks it could be adapted to other biopolymers with similar behavior, such as collagen. Other authors have proposed continuous approaches different from WLC [24,33]. Discrete models that simulate the biopolymers filamentous structures using various lattice methods have been widely used due to their ability to reproduce network geometry [26,28,34–39]. Molecular dynamics has been recently used to model collagen fibrils and crosslinkers to investigate their mechanical properties [40–42].

In this work we present a combined experimental and computational study of collagen hydrogels rheology. We have analyzed the mechanical implications of varying the collagen-based concentration and adding a crosslinking agent to collagen hydrogels. We have also used a continuum computational model to improve the understanding of the mechanical behavior of collagen networks and their relationship with network architecture. In fact, the main advantage of this model in comparison with others is that, when we compare with the rheological measurements, we can numerically estimate three parameters with a relevant biophysical meaning (the contour length, the number of fibers per unit volume and the internal pre-stress of the collagen network). And thus, in this way, we can obtain a quantitative estimation of the characteristics of the collagen network.

## Materials and methods

### Rheology

Collagen gels were prepared following the methodology proposed by Shin et al. [43] by means of diluting collagen type I (BD Biosciences) with 10xDPBS (Lonza) and cell media (HOB) in the required amounts to obtain the desired collagen concentrations. The pH of the dilutions was adjusted to 7.4 by adding 0.1M NaOH. The final collagen concentrations were 1.5, 2.0, 2.5 4.0 and 6.0 mg/ml, which are usually used in cell culture experiments [44–47]. Additionally, purified recombinant human Transglutaminase II (TG2) in solution (R&D Systems) was added, at a final concentration of 25μg/ml, to hydrogels to investigate whether the crosslinking agent modifies the mechanical behavior of the hydrogels. We studied the effect of the crosslinking agent on three hydrogels with higher collagen concentration, that is, 2.5, 4.0 and 6.0 mg/ml. Low collagen concentrations have not been crosslinked, because when low collagen gels are used in microfluidic experiments, cells are able to loose the gel from the chamber in the microfluidic chip.

Collagen hydrogels were characterized by rheology to evaluate their mechanical properties using a stress-controlled (Haake Rheostress 1) rheometer. All samples were tested using a cone-plate configuration with a 35mm diameter and a cone angle of 1°. During the measurements the rheometer applies an oscillatory shear stress, *τ* = *τ*_0_ sin(*ωt*) and the resulting shear deformation γ is quantified.

**Fig 1:**
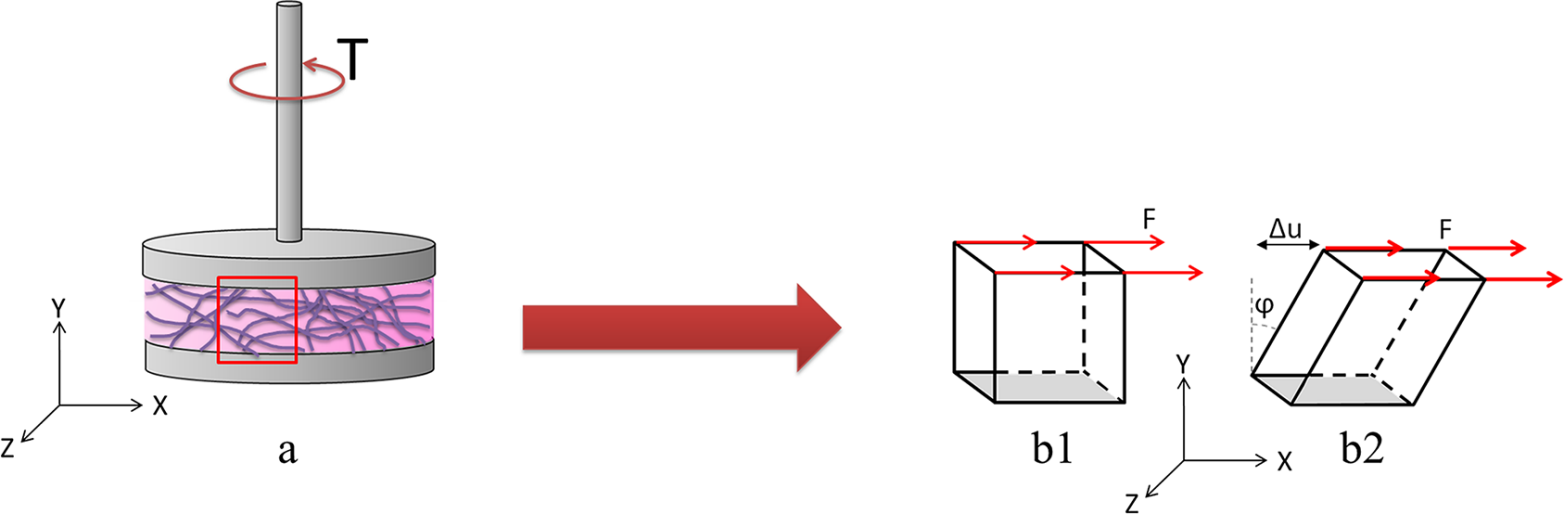
Experiment scheme. a) Scheme of the hydrogel in the rheometer in the experiment. b) Equivalent geometry used in the computational simulations in the undeformed state (b1) and after loading (b2).

For each collagen hydrogel the sample was prepared and immediately placed on the lower plate of the rheometer. Next, the upper plate descended until the gap between both plates was the required by the sensor specifications (0.051 mm) (Fig 1A). As a result of the gel confinement a small pre-stress is induced on the hydrogel sample. The sample contour was covered with low viscosity oil (0.1 Pa·s) to prevent dehydration during the experiment. The samples polymerized in-situ during 24 hours at 37°C and under a cyclic strain of amplitude 0.5% and frequency 0.1Hz. During this time, the storage shear modulus (G’), which describes the material's elastic response to shear stress, was instantly evaluated. G’ is a measure of the energy stored in a material when a deformation has been imposed. It represents the proportion of the total rigidity that is due to elastic deformation. On the other hand, the loss shear modulus, G”, is a measure of the energy dissipated in a material during a deformation. It refers to the proportion of the rigidity of the material that is due to viscous flows, rather than elastic deformation. The dynamic viscosity denoted by η is a measure of the resistance of a material to flow.

After the polymerization period gels were mechanically loaded applying cyclic oscillatory stress sweeps with an excitation frequency of 0.1 Hz. The rheometer was stress controled and the initial applied stress was in each case the smallest allowed by the rheometer specifications, a minimum torque of 5 μN·m which generates a minimum stress of 0.446 Pa for the applied geometry. The stress was increased in each cycle until failure. The application of the minimum torque gives a starting strain that varies depending on the hydrogel mechanical properties. The complete process, including hydrogel preparation, was repeated three times for each hydrogel composition. Bands were constructed for the three curves of each experiment, in order to verify that these testing repetitions can be considered equivalent. In fact, we observed that the standard deviation is always approximately less than 10% of the mean value. Nevertheless, further measurements would be necessary for a full statistical analysis.

### Computational model

In addition to the experimental work we have implemented a computational method that allows us to estimate the structural properties of hydrogels based on hydrogel composition. The model reproduces the stress sweep analysis as performed in the rheological work.

To evaluate the shear modulus of collagen hydrogels we have followed the numerical approach proposed by Palmer and Boyce [29]. Although they simulate actin networks their model can be applied to collagen hydrogels because both materials present a similar mechanical behavior. Palmer and Boyce developed a new filament-force relationship (Eq 1) from the one proposed by MacKintosh [31] and combined it with the Arruda-Boyce eight chain model [32]. The worm like chain (WLC) model allows to evaluate the force-extension relationship of semiflexible individual filaments while the eight-chain model accounts for the network structure. The model characterizes hydrogels through several parameters but in this work we have focussed only on three parameters: the contour length (*L*_*c*_), the number of fibers per unit volume (*n*) and the network pre-stress, defined through the percent increase of the initial end-to-end distance (α). The contour length refers to the length of filaments between network junctions when they are completely extended [29].

The model is defined by the force-extension relationship proposed by Palmer and Boyce (Eq 1) and the strain energy function of the eigth-filament model (Eq 2)

$$F_{Mac}=\frac{k_{B}T}{l_{p}}\left(\frac{1}{{4(1-r/L_{c})}^{2}}\right)\left(\frac{L_{c}/l_{p}-6(1-r/L_{c})}{L_{c}/l_{p}-2(1-r/L_{c})}\right)$$ (1)

$$\psi_{Mac}=\frac{{nk}_{B}T}{l_{p}}\left[\frac{L_{c}}{4(1-r/L_{c})}-l_{p}\left[ln({L_{c}}^{2}-2l_{p}L_{c}+2l_{p}r)-ln(r-L_{c})\right]-c\right]$$ (2)

where *k*_*B*_ is the Boltzmann’s constant, *T* is the absolute temperature, *c* is the initial strain energy density from the filaments, l_p_ is the persistence length, the length over which filaments appear straight in the presence of Brownian forces [11] and *r* is the end-to-end distance. The end-to-end distance, *r*, evolves as long as the chains are stretched,

$$r=\lambda_{c}r_{0}$$ (3)

Where *λ*_*c*_ is the strecth of any chain and *r*_*0*_ is the initial end-to-end distance, which is determined from the structural parameters and the network prestress

$$r_{0}=L_{c}\left(1-\frac{L_{c}}{6l_{p}}\right)(1+\alpha )$$ (4)

A more complete description of this model can be found on the work by Palmer and Boyce [29].

In this work we simulated a cubic geometry equivalent to an infinitesimal portion of the sample in the rheometer (Fig 1). The base of the cube was fixed while the upper face was cyclically displaced reproducing the experimental oscillatory test (Fig 1B1 and 1B2). A displacement gradient is created in the gel geometry, the base stays fixed while the top moves parallel to the base.

## Results

### Rheology

#### Collagen concentration regulates stiffness during polymerization

**Fig 2:**
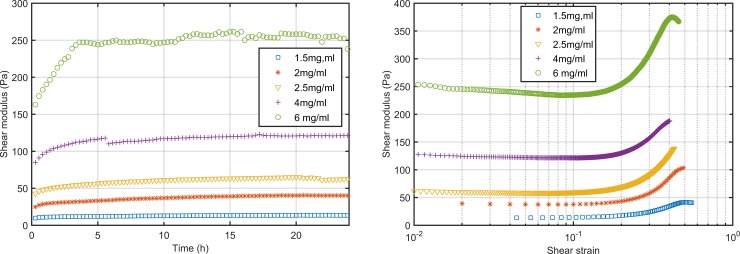
Storage shear modulus during polymerization of collagen hydrogels. Polymerization of five hydrogels with different collagen concentration without TG2 (left). Stress sweeps of five collagen hydrogels with different collagen concentration (right) after 24 hours of polymerization. Three independent specimens were analyzed for each hydrogel composition.

**Fig 3:**
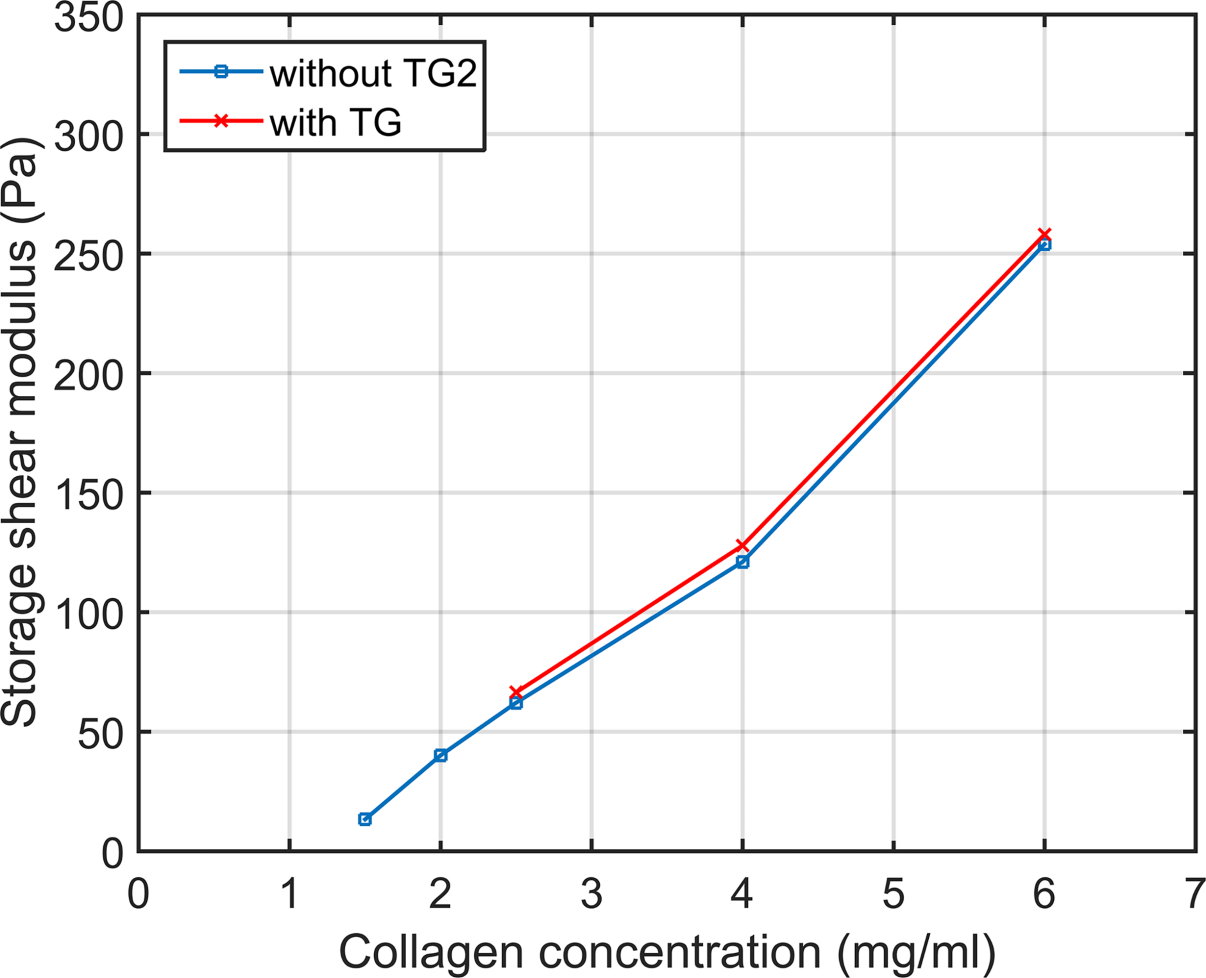
Storage shear modulus of collagen hydrogels with varying collagen concentration after polymerization, with and without TG2.

The evolution of the gel stiffness was evaluated during 24 hours of polymerization. The shear modulus (G’) highly increased during the first five hours and more slightly during the rest of the process (Fig 2, left). As expected, the shear modulus value at the end of the polymerization was higher as the collagen concentration increased. We found that the shear modulus of the polymerized gels presents a linear dependence on the collagen concentration within the studied range, from 1.5 mg/ml to 6 mg/ml (Fig 3). We have observed that, in general, the variability of the measured shear modulus increases as long as gels are stiffer (Table 1).

**Table 1:** Storage shear modulus(G’), lossmodulus(G”), dynamic viscosity (η’) and loss viscosity (η”) of collagen hydrogels with different composition after polymerization.

Collagen concentration	G’(Pa)	G”(Pa)	η’ (Pa∙s)	η” (Pa∙s)
1.5	13.35 ± 0.36	1.37 ± 0.11	2.19 ± 0.17	21.25 ± 0.57
2.0	40.12 ± 3.29	3.43 ± 0.33	5.45 ± 0.53	63.86 ± 5.24
2.5	62.14 ± 4.87	5.00 ± 0.39	7.96 ± 0.62	98.90 ± 7.75
2.5 + TG2	66.46 ±6.29	4.60 ± 0.28	7.32 ± 0.45	105.78 ± 10.02
4.0	121.03 ± 9.94	11.57 ± 0.58	18.42 ± 0.92	192.63 ± 15.82
4.0 + TG2	127.90 ± 14.43	11.10 ± 0.16	17.45 ± 0.26	203.56 ± 22.96
6.0	254.05 ± 29.06	24.60 ± 0.28	39.15 ± 0.45	404.19 ± 32.71
6.0 + TG2	258.05 ± 3.89	16.2 ± 0.16	25.78 ± 0.26	410.70 ± 6.19

We have also included in Table 1 the mean value of the loss modulus (G”) which represents the viscous contribution, the dynamic viscosity (η’) and the storage viscosity (η”). We observe that the loss modulus, which denotes the energy loss, increases with the collagen concentration, in a proportion similar to the incensement of G’. Nevertheless, when TG2 is added the shear modulus is slightly lower than in the absence of TG2. Storage viscosity (η”) and dynamic viscosity (η’) are respectively related to G’ and G” through the angular frequency (ω) applied to the sample. Thus, they present a similar evolution to G’ and G” when the hydrogel composition changes. Nevertheless, in all cases, G' is higher than G'', which implies that collagen-based hydrogels present a predominantly solid rather than fluid-like behavior.

#### Collagen concentration regulates the elastic behavior

The second part of the characterization consisted on the measurement of the shear modulus during an oscillatory stress sweep assay. Frequency was fixed at 0.1Hz for all the measurements. The initial strain, for the minimum allowed torque, depends on the gels properties. The initial shear modulus value registered was close to the one measured during the final stage of the polymerization at small strains. Stiffer hydrogels allowed a lower initial strain than softer hydrogels. From a strain around 15% all the tested hydrogels began to show strain-stiffening (Fig 2, right). It is interesting to observe that before strain-stiffening there is a small strain-softening stage, more visible when the collagen concentration is higher. As long as the strain was increased the shear modulus stabilized reaching a maximum value during a short time and then decreased. Finally, we have analyzed the maximum shear strain that hydrogels bear before failure and the maximum G’ measured (Table 2).

**Table 2:** Maximum shear strain before failure (%), maximum G’ during the stress sweeps and strain at the maximum G’.

Collagen concentration	Maximum Shear strain	Maximum G’ (Pa)	Strain at the maximum G’
1.5	0.554	41.5	0.514
2.0	0.490	103.5	0.490
2.5	0.429	138.1	0.429
2.5 + TG2	0.592	314.1	0.510
4.0	0.403	191.2	0.403
4.0 + TG2	0.449	285.5	0.384
6.0	0.458	375.0	0.413
6.0 + TG2	0.568	496.2	0.506

#### The addition of a crosslinking agent delays polymerization

**Fig 4:**
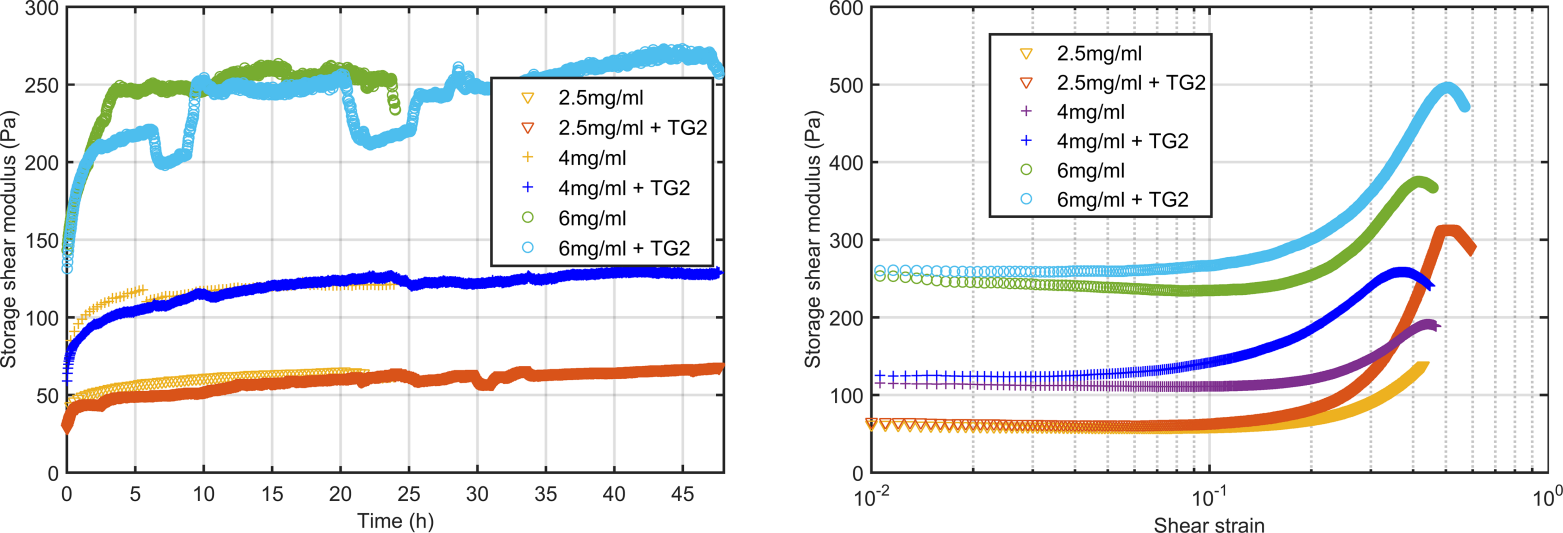
Effect of transglutaminase on the storage shear modulus. Average shear modulus during polymerization of 2.5, 4 and 6 mg/ml collagen hydrogels w/o transglutaminase (left). Evolution of G’ during stress sweeps of the same hydrogels (right). Three independent specimens were analyzed for each hydrogel composition.

In this work we have used transglutaminase, TG2, an enzymatic crosslinking agent, to increase the mechanical properties of the hydrogels. To prepare the new hydrogels we followed the same process as detailed before substituing part of the medium by tissue transglutaminase (TG2) and preserving the total hydrogel volume and the collagen concentration. We added TG2 to the three stiffer hydrogels, the ones with 2.5, 4 and 6 mg/ml of collagen, to obtain even stiffer hydrogels. In those cases we observed that the shear modulus kept increasing after 24 hours and the complete polymerization took place later than in the absence of TG2 (Fig 4A). This phenomenon was observed in the three crosslinked hydrogels. Thus, we increased the polymerization time up to 48 hours, when we observed that the shear modulus was almost constant.

Within the collagen concentration range that we investigated it is found that the shear modulus varies linearly (r^2^ = 0.9944) with the collagen concentration (Fig 3). This phenomenon was also visible when we added TG2 to the collagen gels (r^2^ = 0.9852), finding that the values in presence of TG2 are slightly higher. In the case of 6mg/ml hydrogels with TG2 we observe jumps in the shear modulus probably caused by environmental or measurement conditions. However, we observe that the polymerization tendency is not modified.

#### Crosslinked gels present a stronger strain-hardening phenomenon

After the 48-hours polymerization we performed the stress sweep cycle test. Following the same procedure as for the hydrogels without transglutaminase we applied cyclic loads to the sample inducing an increasing shear strain starting from the minimum possible strain.

For the three collagen concentrations we observed a similar behavior (Fig 4B). For the transglutaminase concentration used, the shear modulus is slightly higher in the low deformations range. For higher deformations, we observed earlier stiffening in both cases, stiffening began for smaller strains when TG2 was added. It is also important to note that the strain- softening stage observed in the absence of TG2 never occur when we added the enzyme.

We also observed differences in the failure stress, even though the maximum strain that hydrogels bear before failure is higher when the transglutaminase is added (Table 2). Moreover, the stress that they bear is higher in crosslinked gels, which is translated into higher shear modulus values.

### Network structure can be estimated with computational simulation

We have used the computational model presented in Section 2.2 to predict the evolution of G’ during the application of stress sweeps. The model characterizes the hydrogels through three main structural parameters: the contour length (L_c_), the number of fibers per unit of volume (n) and the network pre-stress, defined through the percent increase of the initial end-to-end distance (α). As a result of the hydrogel composition these parameters modify their value and thus, it is possible to reproduce the behavior of any gel. Inversely, it is possible to estimate the value of these parameters adjusting simulations to experimental measurements. This method allows characterizing the network structure and the pre-stress level by means of a simple fitting process.

We have estimated the value of structural parameters for hydrogels with and without the crosslinking enzyme TG2 finding significant differences (Table 3). To obtain these values we have used artificial neuronal networks techniques [48,49] using as starting values some obtained from the literature. Regarding the contour length we have observed that in the absence of transglutaminase, hydrogels with higher collagen concentration increase their contour length or their number of fibers, or both. The reason is that as long as the collagen concentration is higher it forms more fibers which are more compressed. Nevertheless, when we add the crosslinking enzyme, the number of fibers still increases respect to the same concentrations without the enzyme and the contour length maintains or takes lower values. One explanation could be that adding the crosslinking agent created more union points, which provides more but shorter filaments, as the collagen concentration remains constant. The model has also predicted that the pre-stress of the crosslinked networks is smaller when we add TG2, probably because smaller fibers can accommodate easier than longer ones to the available space. The value of all the adjusted parameters is presented on Table 3.

**Table 3:** Structural parameters estimated from the rheological behavior of the collagen networks.

Composition	Contour length, L_c_ (μm)	Fiber density, n (fibers/m^3^)	Prestress, α
1,5	26	8.33e21	0.032
2	26	1.83e22	0.032
2.5	26	3.16e22	0.032
2.5 + TG2	20	4.17e22	0.015
4	30	4.66e22	0.060
4 + TG2	20	5.17e22	0.025
6	30	5.64e22	0.060
6 + TG2	30	6.58e22	0.060

**Fig 5:**
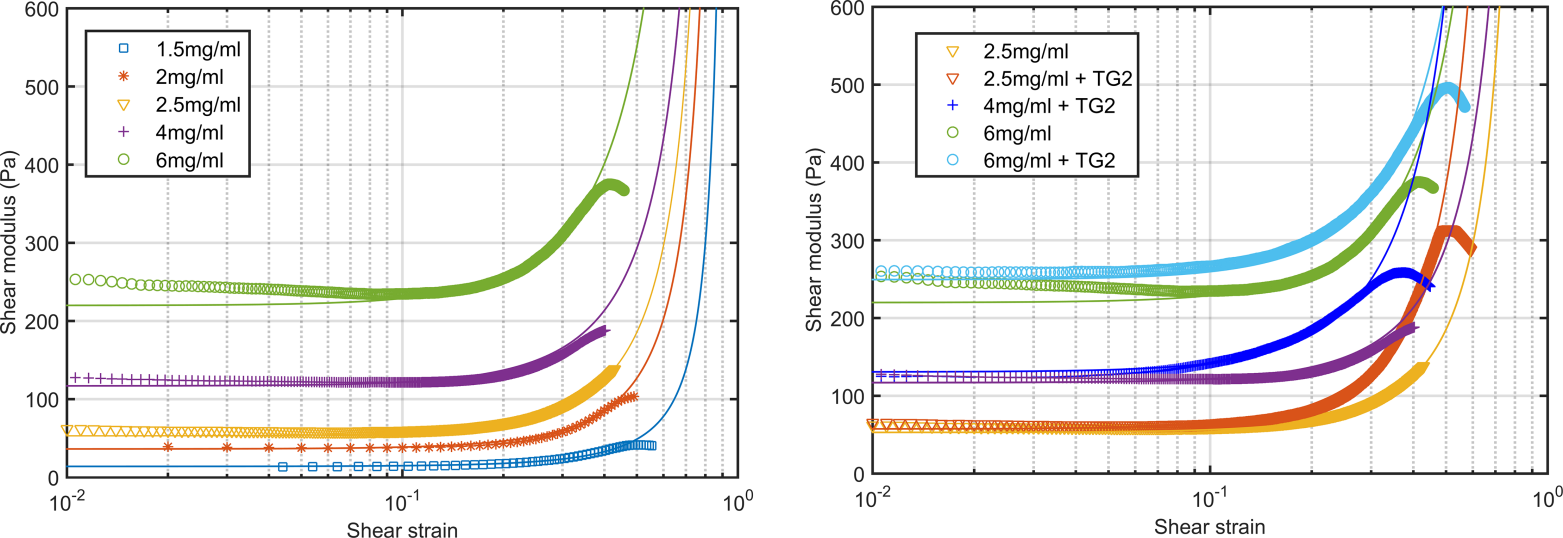
Computational predictions. Experimental measurements and computational predictions of G'(Pa) for 1.5, 2, 2.5, 4 and 6 mg/ml hydrogels without transglutaminase (left) and for 2.5, 4, 6 mg/ml hydrogels with transglutaminase (right). Experimental results are showed by symbols and computational predictions are denoted by continuous lines.

Finally, we compare the experimental results and the best fitting prediction obtained with the computational model (Fig 5). We observe that the model does no predict the strain-softening observed without the TG2 and thus it fits better crosslinked hydrogels.

## Discussion

We have presented an experimental study of the effect of collagen hydrogels composition on its mechanical properties. We have focused on the hydrogels stiffness and nonlinear elastic behavior when the collagen concentration is varied and when a crosslinking enzyme (transglutaminase) is added to the gels. We have studied five different collagen concentrations and the effect of TG2 on the three gels with the higher collagen concentration. Although the effect of collagen concentration has been recently studied [16,26,50,51] we have not found any study that extensively investigates the effect of transglutaminase, the crosslinking agent here studied. In addition, we have used an existing computational model, the worm-like chain model (WLC), to indirectly characterize the mechanical behavior of these biopolymer networks.

The hydrogels here characterized have been previously used to culture fibroblasts [45,46], osteoblasts [47] and endothelial cells (HUVEC) [44]. Processes such as cell migration or angiogenesis have been proved to evolve correctly using these gels and cell viability was confirmed in all cases.

Regarding the experimental study we have analyzed two groups of hydrogels, with and without adding a crosslinking agent. In first place we analyzed collagen gels without altering their structure with crosslinking agents and we studied the polymerization process and the effect of applying increasing loads. During polymerization we have observed that in the absence of TG2 the shear modulus stabilizes, depending on the composition, after around 15 hours, in agreement with the observations on the linear modulus of Roeder et al. [7]. The main effect of increasing the collagen concentration was the increase of the hydrogel stiffness. When the collagen concentration is higher, more fibers are formed and they are more densely packed resulting on a stiffer material [9]. We found a linear dependence of the storage shear modulus with the collagen concentration within the tested range. Thus, it is possible to prepare hydrogels with a desired G’ depending on the requirement of the application. During the application of cyclic loads (Fig 2) we found that more concentrated gels are stiffer, as expected. This fact has major implications for many biological processes, such as cell migration and differentiation, meaning that an excessive collagen production may cause an excessive rigidization of the tissues and that may alter the regular cell behavior. Moreover, collagen hydrogels showed strain stiffening as the deformation that they bear increases, independently of the collagen concentration. This non-linear behavior has been widely described in soft biological tissues [52]. While shear strain remains under 10% the shear modulus remains invariable and when a deformation around 30% is reached the variation of the gels shear modulus is around 150%. Nevertheless, when we increase the deformation over 30% there is fast stiffening, reaching a shear modulus up to twice the initial value (Table 2). An interesting phenomenon was observed when the strain was increased at the beginning, hydrogels showed a small relaxation and a decrease of 5–11% on the shear modulus value. This phenomenon was consistent with the observations of Kurniawan et al. [16]. Nevertheless, this phenomenon has not been deeply studied in other works. Kurniawan et al. [16] proposed that the strain-softening could be a result of the redistribution of internal stresses due to the slipping of the physical crosslinks. Moreover, when we add TG2 to the gels we do not observe the softening stage. We hypothesize that, as enzymatic crosslinks are stronger than physical crosslinks, even if there is slippage of the physical crosslinks the enzymatic ones remain fixed and there is no visible relaxation. Moreover, softening is due to the crimped nature of collagen fibers. Collagen fibers are initially relaxed and while the hydrogel is deformed fibers are straightened. While they are not stretched over their contour length they do not oppose to material deformation and thus stiffness does not increase. Finally, if we compare G’ and G” we observe that for all the hydrogels G’ is one order of magnitude greater than G”, which means that the elastic component has higher influence than the viscous component and that more energy is stored than dissipated.

To increase the mechanical properties of collagen hydrogels we added transglutaminase as a crosslinking agent. In the literature, we can find different crosslinking methods that can be classified into chemical, physical and biological [53]. The mechanism, capacity and efficiency of each crosslinking is different and is crucial in the final mechanical properties of the collagen-based scaffolds as was determined by Zeugolis et al [53]. Most of the chemical and physical crosslinking methods [2,15,16,54] may present some levels of cytotoxicity for cell culture experiments, therefore, we have mainly focused on one biological crosslinking system, such as the transglutaminase. Actually, there are many different previous studies [21–23] that analyze the effect of transglutaminase on collagen-based gels, although none of them focuses on their mechanical behavior.

We observed that when transglutaminase is added the main effect is observed at high deformations. Actually, the gel stiffness is not notably modified when small deformations are applied; we observed that as long as we increased the strain the stiffness also increased earlier than in the absence of TG2. Whether the variation of the transglutaminase concentration would affect this behavior needs further investigation to have a complete view of the crosslinking effect, although Fortunati et al. [23] postulates that the enzyme is unable to increase gel rigidity when the collagen concentration is above a limit due to the high fibrils density. This observation is corroborated by the results obtained in this work. We observe that the TG2 effect is less appreciable when the collagen concentration is higher. However, when large oscillation loads were applied we found that the effect of transglutaminase was higher in those hydrogels with the higher collagen concentration. In any case, we have to keep in mind that the analysis here presented is a first analysis of how one enzymatic crosslinker regulates mechanical behavior of collagen-based hydrogels. Certainly, our work presents some limitations, because in order to achieve a full characterization of our collagen-based gels, it would be necessary to carry out some additional biochemical and enzymatic degradation analysis. In addition, we have focused our analysis in only one enzymatic crosslinker, when many other different strategies for crosslinking collagen gels could have been used, such as those proposed by Sanami et al and Zeugolis et al [53,55]. In future works, we are going to extend our work to understand the impact of other crosslinkers in the mechanical behavior of collagen-based gels when they are used for in-vitro cell cultures.

This work has been complemented with numerical simulations of the corresponding experiments. One advantage of computational models is that properly adjusted they can be used to simulate networks made of different biopolymers and compositions. Inversely, it is possible to estimate the value of the network parameters adjusting simulations to experimental results. This method allows characterizing the network structure without the necessity of more sophisticated methods such as image processing or network reconstruction. Nevertheless, we have to keep in mind that due to the limitations of our rheometer only shear measurements were used to adjust the numerical model. In other more modern rheometers normal stresses can be quantified, which would allow to improve the fitting process of the mechanical parameters.

From our computational results, we observe that adjusting properly the parameters value, the predictions are closer to experimental results as long as the collagen concentration is lower (Fig 5). Moreover, in the absence of TG2 there is always a smooth softening prior to stiffening which is not appreciable for low collagen concentrations. Our WLC model is able to accurately predict this strain stiffening, although it cannot simulate softening when collagen concentration is increased. Nevertheless, simulations provide an accurate prediction about the shear value at low strains and the strain that initiates strain-stiffening, which is even more accurate in the prediction of crosslinked hydrogels. Finally, the parameters here estimated together with the WLC model can be applied to complex simulations where the nonlinear behavior of the extracellular matrix plays an important role.

The role of the mechanical and structural properties of the extracellular matrix on the cellular biological response has been extensively studied [3,4,56–58] due to their relevance on crucial processes such as cell proliferation, migration [59–61], differentiation [62] and apoptosis [63].

Many different aspects have been analyzed, but especial emphasis has been focused on the dependence of the migration model on matrix elastic behavior [3,4,64]. In fact, the elastic behavior of the ECM in part governs 3D migration, distinguishing between lamellipodial and lobopodial migration [64]. Lobopodia-based migration occurs in pure linear elastic matrices, lamellipodia in nonlinear elastic materials. Therefore, it is important to detect differences between the linear and nonlinear elastic behavior of collagen based hydrogels. From our results, we have demonstrated that in pure collagen-based hydrogels the nonlinear elastic behavior is nondependent of the collagen concentration. However, when these gels are crosslinked with TG2, nonlinear elastic response appears to lower strains. This fact is crucial in many pathological conditions such as hepatocellular carcinome [63] or myocardial fibrosis [65] where enzymatic crosslinkers may stiffening by strain hardening inducing an anomalous nonlinear elastic behavior of the tissue.

The possibility of creating collagen hydrogels with the desired mechanical properties makes them suitable for a large number of applications. Collagen hydrogels are broadly used in cell cultures. It is known that cells behave differently depending on their mechanical microenvironment and hydrogels offer the possibility of selecting their mechanical properties and observe their effect on cellular response. Another promising field in which biopolymeric hydrogels can be applied is tissue engineering. Due to their tunable properties hydrogels can be used to mimic biological tissue and for organ fabrication [66–69]

## Conclusions

The mechanical properties of collagen hydrogels depend on many factors, mainly the hydrogel composition, the preparation method and the environmental conditions. In this work we have focused on understanding the mechanical role of collagen concentration and enzymatic crosslinking on the hydrogel stiffness and elastic behavior. Differences have been found on the polymerization process and on the response under cyclic loads when the composition is varied. Regarding the collagen concentration we have found a linear relationship between the concentration and the gel stiffness, together with an earlier stiffening of more concentrated hydrogels. In addition, TG2-mediated crosslinking does not increase highly the shear modulus at small strains but promotes earlier stiffening. Finally, the nonlinear elastic behavior of the collagen hydrogel can be predicted or adjusted with the computational approach presented, allowing to understand the relationship between the biopolymer network structure and its mechanical properties.
